# Preface

**DOI:** 10.2174/138920292401230610190952

**Published:** 2023-06-23

**Authors:** Christian Neri

**Affiliations:** 1 Research Director at INSERM, Brain-C Lab, Institute of Biology Paris-Seine

Current Genomics publishes peer-reviewed articles, including review and original articles that cover the last developments and recent advances in the field of genome sciences. Articles about the added value of genome sciences to understanding evolution, development, aging, and plant biology, as well as epigenetic regulation, molecular biology of intercellular communication, cell identity and homeostasis, and human diseases, are of primary interest to Current Genomics, especially if they contain a strong component in systems biology or systems modeling. The Editorial Board of Current Genomics is greatly looking forward to continued work and interactions with the community of experts in genome sciences to publish high-quality data that address key challenges in biology and translational research.

Best Wishes,

Christian

